# SARS-CoV-2 Anti-Spike IgG Subclass Titres in a Population with Prior Exposure to Perfluorooctanoic Acid (PFOA)

**DOI:** 10.3390/toxics14030192

**Published:** 2026-02-25

**Authors:** Mihai Zamfir, Doris Gerstner, Heidi Lahne, Volker Fingerle, Johannes Redwitz, Wolfgang Schober, Bettina Aschenbrenner, Matthias Graw, Dennis Nowak, Caroline Quartucci, Caroline Herr, Stefanie Heinze

**Affiliations:** 1 Bavarian Health and Food Safety Authority, Institute for Occupational Health and Product Safety, Environmental Health, Pfarrstr. 3, 80538 Munich, Germany; 2 Bavarian Health and Food Safety Authority, National Reference Center for Borrelia, Veterinärstr. 2, 85764 Oberschleissheim, Germany; 3 Institute of Forensic Medicine, Ludwig-Maximilians-Universität in Munich, Nussbaumstr. 26, 80336 Munich, Germany; 4 Institute and Clinic for Occupational, Social and Environmental Medicine, University Hospital, LMU Munich, Ziemssenstr. 5, 80336 Munich, Germany; 5 Comprehensive Pneumology Centre Munich (CPC-M), German Centre for Lung Research (DZL), 81377 Munich, Germany; 6 Pettenkofer School of Public Health, Ludwig-Maximilians-University Munich, Elisabeth-Winterhalter-Weg 6, 81377 Munich, Germany

**Keywords:** PFOA, SARS-CoV-2, IgG

## Abstract

The role of perfluorooctanoic acid (PFOA) on antibody titres following vaccination in adults is not heterogeneous. High levels of anti-spike IgG have been reported following mRNA-based vaccinations. This study aimed to explore the association between PFOA exposure and exposure to SARS-CoV-2 antigen on SARS-CoV-2 anti-spike antibodies, particularly on the IgG subclasses in adult residents with increased internal PFOA exposure due to past PFOA drinking water contamination. A self-administered questionnaire was completed, and vaccination data was checked against the vaccination passport. PFOA concentration and IgG subclass titres were analysed in serum. Most study participants had three vaccinations (518; 78.6%). IgG was dominated by IgG4, a subclass that is largely unable to activate effector responses, for 8.3% of the participants, and only in the group of participants with three vaccinations. A generalised linear model was used to assess the relationship between PFOA serum concentration and IgG subclasses. After adjusting for age, a significant positive association was found between the number of vaccinations, self-reported infections and higher IgG subclasses concentrations. Age and PFOA concentration did not show a significant association with the concentration of any IgG subclass. Thus, the internal PFOA exposure was not associated with impaired immune response with regard to anti-spike SARS-CoV-2 IgG.

## 1. Introduction

As part of the strategy to limit the spread of SARS-CoV-2 and prevent severe COVID-19 infections, multiple vaccine platforms were developed rapidly, and many countries, including Germany, used mass vaccination strategies. The vaccines exploit the S-protein antigen, except for those with inactivated virus, and apply multiple dose schemes, usually 2 immunisations within 4 weeks followed by additional booster vaccinations [1,2].

Following exposure to SARS-CoV-2 S-protein antigen, either by infection or vaccination, the adaptive immune system develops antigen-specific antibodies, such as Immunoglobulin G (IgG), which is the principal anti-SARS-CoV-2 antibody [3,4,5,6]. Once bound to an antigen, IgG can bind to different effectors with the Fc domain and thus facilitate phagocytosis, T-cell stimulation, activation of the complement system via C1Q, and antibody-dependent cytotoxicity [7,8,9]. Anti-SARS-CoV-2 IgG antibody level has been shown to be a good protective marker [6,10,11,12,13].

In natural infections, IgG anti-SARS-CoV-2 is mainly formed against the S and N proteins, a week after the onset of symptoms, usually when there is no more viremia [14,15,16]. Antibodies against membrane and envelope proteins have also been reported [5]. Dan et al. [17] and Yates et al. [18] report that anti-S IgG antibodies have a longer half-life (140–225 days) than anti-N IgG. The increase in IgG titre has been shown to correlate with disease severity [3] and mainly consists of IgG1 and IgG3, where, in the short term, IgG3 is in a higher proportion [5,18,19,20]. IgG2 and IgG4 have only been reported in a small number of participants by one study [18].

High levels of anti-S IgG have been reported following mRNA-based vaccines [4,21,22,23,24], with a similar subclass distribution, marked by high levels of IgG1 and IgG3 and low levels of IgG2 and IgG4, as other SARS-CoV-2 vaccines or natural infections [23,25,26]. At one month after a booster dose, a 1.8 to 3.3-fold increase in antibody titre compared to one month after primary immunisation was observed [27,28]. An even higher increase in IgG titres was also reported by heterologous vaccinations [1]. Increased age (over 60) has been reported to reduce both the IgG titre and the subsequent activation of effectors [29,30].

In vaccinated individuals, mRNA-induced S-protein production could be detected more than 57 days after the last vaccination [31]. IgG3 titres decline relatively rapidly (100–150 days), and IgG4 titres increase over time [21,25,32]. IgG4 levels are usually elevated after repeated or continuous exposure to antigens, to allow immune tolerance and a weak Fc-mediated response due to its low affinity for Fc receptors [33]. Several studies report that repeated vaccination increased the concentration of non-inflammatory IgG4 and increased the IgG4/IgG1 or IgG4/total IgG ratios [21,25]. The contribution of IgG4 to the total IgG pool seems to increase with time. Irrgang et al. [21] reported an increase even after 180 days after the third vaccination, reaching 40–80% of the anti-S-antibodies. A similar increase in IgG4 titre was not found after vaccination with adenoviral vectors [21,25,32]. Currently, this phenomenon has not been fully understood, with some hypotheses regarding the persistence of vaccine mRNA or antigens in lymph nodes determining the immune system to switch to non-inflammatory IgG4 antibodies [31,34]. High IgG4 levels have been associated with lower potential to activate Fc-mediated effector functions, weak antibody-dependent cellular phagocytosis (ADCP) and complement activation compared to IgG1 and IgG3 [21,25]. When looking into antigen exposure by infection, the IgG4/total IgG ratio and IgG4/IgG1 ratio were found to be significantly higher in non-survivors of COVID: a concentration above 700 mg/dL serum IgG4 and an IgG4/IgG1 ratio above 0.05 were significant predictors of 30-day mortality [35].

Perfluoroalkyl substances (PFAS) are persistent environmental contaminants with a high potential to bioaccumulate and are considered to cause immune suppression [36,37]. Environmental exposure to some PFAS, such as perfluorooctanoic acid (PFOA), has been linked with adverse health outcomes, such as lower antibody concentration in children following vaccination against tetanus and diphtheria [38,39], and in adults, lower cardiovascular, kidney, liver and thyroid function. Examination of the relationship between PFOA and IgG isotype in newborns found a positive association with IgG2; however, no clear effect on their health status is mentioned [40]. In German infants, PFOA concentration was significantly associated with lower response to tetanus and diphtheria vaccination [36]. Studies in an adult population did not find consistent results on the effect of PFAS on the immune system [41]. In an adult population, Looker et al. [42] found no association between PFOA serum concentration and the influenza anti-A/H3N2 IgG antibody titre rise after adjusting for age, nor with the influenza B antibody titre rise. The relation between workplace PFAS exposure and SARS-CoV-2 antibodies was examined by Porter et al. [43], where they found small inverse associations for perfluorooctane sulfonic acid (PFOS), PFOA, perfluorohexane sulfonic acid (PFHxS) and perfluorononanoic acid (PFNA). A higher risk of COVID-19 was associated with higher urinary concentrations of PFOS and PFOA, as reported by Ji et al. [44]. Similarly, a Bayesian ecological regression model of a PFAS-contaminated region in Italy detected a rate ratio of 1.6 [90% Credibility Interval: 0.94; 2.51] for the crude mortality rate from COVID-19 [37]. Bailey et al. [41] did not find any association between PFOA and anti-S IgG antibodies either immediately after vaccination or in the study’s follow-up time.

While production and use of PFOA is no longer permitted in the EU [45], the district of Altötting, Bavaria, Germany, was contaminated with PFOA from 1968 until 2003 from a production facility. Contaminated drinking water was considered the main PFOA exposure source for the general population, with high levels of PFOA having been detected. Active carbon filtration for the drinking water systems were put in use since 2007. A previous study in this district analysing serum samples from 2018 found highly elevated levels of PFOA in serum, above the levels where adverse health effects are to be expected (Human Biomonitoring I; HBM-I) for many participants [46]. In comparison to a control group from Munich, Germany, PFOA was the only PFAS statistically different in the Altötting district. A follow-up study, after the half-life period, found a lower PFOA serum concentration in this population [47].

This study explored the potential association between PFOA exposure and repeated exposure to SARS-CoV-2 antigen on SARS-CoV-2 antibodies, particularly on the IgG subclasses, in adult participants from the Altötting district.

## 2. Materials and Methods

Participants in the 2018 human biomonitoring study were invited to take part in the follow-up study in 2022. Further details regarding recruitment and study design have been previously reported [47]. Of the 717 study participants over 18 years old, 55 were excluded as they reported occupational exposure to PFOA or occupational exposure information was not provided. The present study was designed for the general population and was not adapted to determine patterns of work-related PFOA exposure or occupational exposure limits. In addition, three participants were excluded as they did not provide any information on SARS-CoV-2 exposure, either vaccination or infection. Thus, 659 participants were included in the analyses.

Blood samples and questionnaire information were collected between June and August 2022. Self-reported information on vaccination status was checked against the vaccination certificate by a healthcare professional on location and corrected if necessary. After centrifugation, the serum samples were delivered to the laboratory under monitored conditions and kept at 4 °C.

Serum concentrations of SARS-CoV-2 IgG subclasses were quantified using ELISA. Briefly, high-binding plates (Greiner Bio-One GmbH, Kremsmünster, Austria) were coated with 100 ng/well recombinant SARS-CoV-2 spike protein (R&D Systems, Minneapolis, MN, USA) suspended in coating buffer (8.4 g/L NaHCO_3_; 3.56 g/L Na_2_CO_3_ in H_2_O; pH = 9.6). After washing the plates three times with 200 µL 0.05% Tween20 in phosphate-buffered saline (PBS), the plates were incubated for one hour at room temperature with 200 µL 5% skimmed milk powder in wash buffer, followed by three more washes with wash buffer before adding the samples. A 2% skimmed milk powder in the wash buffer was used to dilute the serum samples before incubation. The dilution factor was adjusted according to the previously known COBAS values for anti-S SARS-CoV-2 antibodies; samples under 12,000 U/mL were diluted by a factor of 300, and samples over this value by a factor of 1000. After antibody binding incubation for one hour at room temperature, the plates were washed three more times with wash buffer, 100 µL HRP-coupled IgG-specific antibody (SouthernBiotech, Birmingham, AL, USA) and incubated for one hour at room temperature. Finally, the wells were washed three times with wash buffer and two times with PBS and 50 µL ECL-Solution was added, and the luminescence was measured using Victor X4 (Perkin Elmer, Shelton, CT, USA). IgG subtype concentrations were then calculated in GraphPad Prism 6 (GraphPad Software, Boston, MA, USA) with fit/spline and “point to point calculation”. The quantification limits for this protocol for the different IgG subclasses were: 670 ng/L for IgG1, 646 ng/L for IgG2, 73 ng/L for IgG3, and 73 ng/L for IgG4. Concentrations below or equal to the quantification limit were classified as negative, above the quantification limit and below or equal to 100,000 ng/L as positive and above 100,000 ng/L as highly positive.

The IgG4/IgG1 ratio was calculated only when both IgG4 and IgG1 were present and different from 0. An IgG1-dominated ratio was attributed to values below 0.5; a balanced ratio for values between the range [0.5, 2] and for values above 2, IgG4 dominance was considered.

For PFOA measurement by liquid chromatography–mass spectrometry (LC-MS/MS), serum samples were cleaned up, proteins were denaturised by acetonitrile, and further interfering components were frozen out. Further purification was reached using Oasis HLB (Waters, Milford, MA, USA), 20 mm × 2.1 mm, 25 µm trap column, injecting 100 µL sample solution [48]. Gradient elution and a Reprosil-Pur C18 AQ material (Dr. Maisch HPLC GmbH, Ammerbuch-Entringen, Germany), 33 mm × 3 mm, 5 µm, were applied on the UltiMate 3000 (Thermo Fisher Scientific, Waltham, MA, USA) HPLC system in line with a QTrap 5500 (SCIEX, Marlborough, MA, USA) mass spectrometer in negative mode. Calibration ranged from 0.05 µg/L to 20 µg/L, and the limit of quantification was estimated to be 0.25 µg/L for PFOA. Details on standards used, quality criteria and measuring conditions were previously reported [49].

Similar to previous studies [50], the value of 10 µg/L PFOA is used as a cut-off value for the categories not only because it is near the median value in our study population, but it is the value determined by the HBM Commission for adverse health effects in the general population (HBM-II) [51,52].

Descriptive analysis on the PFOA serum concentration, age, gender and number of SARS-CoV-2 antigen exposures was performed in relation to the IgG subclasses. The number of SARS-CoV-2 antigen exposures is presented as a combination of the number of vaccinations and the number of SARS-CoV-2 infections confirmed by PCR. The association between the variables considered for the generalised linear model was checked using Spearman correlation.

Given the small numbers for individual IgG categories and antigen exposures, the measured concentrations of each IgG subclass were used as the dependent variable in a generalised linear model. Because IgG distribution was right-skewed, values were logarithmically transformed. In addition to the exposure variable of serum PFOA levels, several other self-reported variables (age, sex, number of vaccines and infections) were included in the multivariate model. For the reasons mentioned above, and as PFOA was not normally distributed, the categorised PFOA serum concentration was included in the model. Additionally, interactions between PFOA, number of infections, number of vaccinations, days since last exposure (either infection or vaccination), smoking status, medication intake, chronic diseases, age and sex, and models with logarithmically transformed PFOA concentration were calculated. Models with logarithmically transformed PFOA concentration and days since last exposure were included in the Supplementary Materials as a sensitivity analysis for our approach. The statistical analysis was done using SAS 9.4 (SAS Institute Inc, Cary, NC, USA) and significance was reported at α ≤ 0.05.

The Medical Faculty of the Ludwig Maximilian University Ethics Committee provided ethical approval for this study (21-1204 on 25 February 2022), and written informed consent was obtained from all the participants.

## 3. Results

### 3.1. Population Description

This follow-up study included 659 participants. No participants reported diseases related to the immune system at birth or had transplants. The included study participants had an average age of 54 years (SD 14.76), with 57.21% (377) being female. A serum PFOA concentration over 10 µg/L was found in 45.07% (297) of the participants (Table 1).

### 3.2. SARS-CoV-2 Antigen Exposure

Almost all of the participants (609; 92.55%) reported at least one vaccination against SARS-CoV-2, with 78.72% (518) having three vaccinations at the time of the study (Table 1).

At least one previous SARS-CoV-2 infection, confirmed by a PCR test result, was reported by 45.87% (300) of the participants. Many participants (43.27% (283)) reported only one previous SARS-CoV-2 infection, 1.99% (13) reported two previous PCR-confirmed SARS-CoV-2 infections and 0.61% (4) reported three previous SARS-CoV-2 infections (Table 1). When looking at the combined number of antigen exposures, no previous SARS-CoV-2 vaccinations or infections were reported by 2.73% (18). Most of the participants with four antigen exposures had three vaccinations and one infection, 29.59% (195). Of the nine participants with five or more antigen exposures, five (0.76%) reported three vaccinations and two infections, one (0.15%) reported two vaccinations and three infections and three (0.4%) participants reported three infections and three vaccinations.

Age group had a weak significant positive correlation on the number of vaccinations (r = 0.15; *p* = 0.0001), with the participants belonging to a higher age category reporting more SARS-CoV-2 vaccinations, and a mild negative correlation with the number of PCR-confirmed SARS-CoV-2 reported infections (r = −0.28; *p* < 0.0001), with higher age participants reporting fewer infections. The PFOA group was weakly correlated with both gender and mildly with age category.

### 3.3. Anti-S SARS-CoV-2 IgG Antibodies

**Figure 1:**
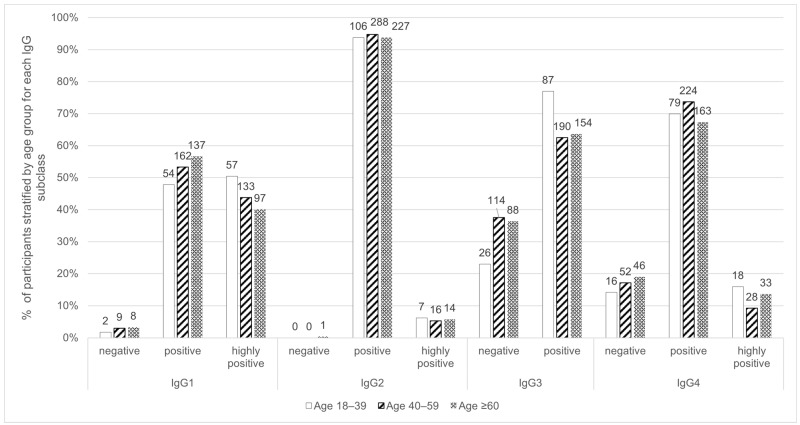
Distribution of IgG subclasses titre categories within the age groups. Bar labels indicate the number of participants.

Almost all the participants were either positive (53.57%; 353) or highly positive (43.55%; 287) for IgG1, positive (94.23%; 621) for IgG2, positive (65.4%; 431) for IgG3 and positive (70.71%; 466) for IgG4. Age category was not significantly associated with the IgG subclass concentration. For IgG3, more participants between 18 and 39 years old had positive values (Figure 1).

IgG1, IgG2, IgG3 and IgG4 levels were significantly positively associated with the number of vaccinations. The IgG4 concentration group had the highest significant correlation with the number of vaccinations (r = 0.51; *p* < 0.0001). More vaccinations were associated with a higher percentage of the participants having positive or highly positive IgG1 and IgG4 concentrations. While the percentage of IgG3-positive participants was higher in the groups with two or more vaccinations, there were no participants with highly positive values. Additionally, in groups with two or three vaccinations, the percentage of participants with IgG4 dominance or a balanced IgG4/IgG1 ratio increased. Of those with three vaccinations, 12.19% had a balanced ratio and 10.25% had an IgG4-dominated ratio (Table 2).

Similarly, the number of self-reported infections was significantly correlated with the concentration of IgG subclasses, where a higher percentage of the participants with one or more reported infection have highly positive IgG1 compared with those who did not report an infection (Table 3). The highest correlation between the number of infections and the IgG subclasses concentration was found for IgG1, at r = 0.34 (*p* < 0.0001). Correlation with IgG4 was the weakest at r = 0.08 (*p* = 0.049). The percentage of the participants in the balanced IgG4/IgG1 ratio category decreased with the number of infections, and the percentage of the participants in the IgG4-dominated IgG4/IgG1 ratio slightly increased between no infection and one infection, from 8.19% (28) to 8.96% (25).

The high number of participants who have positive or highly positive IgG antibody concentrations with no reported infection can likely be attributed to the high percentage of participants with at least one SARS-CoV-2 vaccination. Thus, IgG1, IgG4 and their ratio are also presented against the sum of antigen exposure to SARS-CoV-2, both vaccinations and infections. With an increasing number of antigen exposures, the percentage of participants in the higher categories for all IgG subclasses also increased. IgG4 was mostly negative for the participants with less than three exposures, then mostly positive (Table 4). The correlation between the number of exposures to SARS-CoV-2 and IgG1 concentration category is 0.48 (*p* < 0.0001), and between the number of exposures and the IgG4 concentration category is 0.51 (*p* < 0.0001). IgG3 was also significantly correlated at 0.38, and IgG2 at 0.17. Most participants had an IgG1-dominated IgG4/IgG1 ratio; however, at three and four antigen exposures, 7.61% and 12.76% of the participants had a ratio dominated by IgG4, respectively.

### 3.4. Perfluorooctanoic Acid (PFOA) Serum Concentration

**Figure 2:**
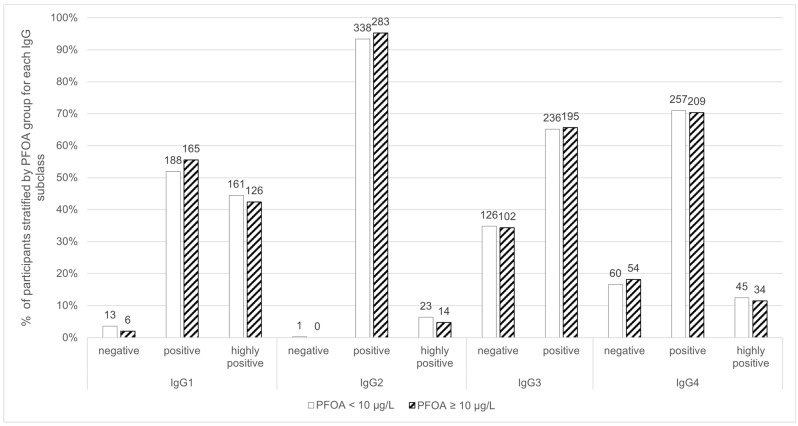
Distribution of IgG1, IgG2, IgG3 and IgG4 titres stratified by PFOA concentration. Bar labels indicate the number of participants.

The study population’s internal PFOA exposure was previously described [47]. Briefly, 45.07% (297) of the study participants had a concentration starting from 10 µg/L PFOA. The PFOA category was significantly moderately correlated with the age category of the participants (r = 0.29), but not significantly correlated with IgG subclass or IgG4/IgG1 ratio. The distribution of IgG1 concentration was similar in both groups, below and above 10 µg/L PFOA (Figure 2). For IgG4, irrespective of the PFOA concentration group, the majority (70.99%; 70.37%) of the participants had positive IgG4 levels, and a similar percentage were highly positive (12.43%; 11.45%) (Figure 2).

In the four regression models with the PFOA serum concentration group (<10 µg/L; ≥10 µg/L) as exposure variable and adjusted for age and sex, the number of vaccines and the number of self-reported infections were positively and significantly associated with anti-S SARS-CoV-2 IgG1, IgG2, IgG3 and IgG4. An additional vaccination was associated with a 5.81-fold (ß = 1.76) increase in serum IgG4 concentration, whereas additional reported infections were associated with a 2.97-fold (ß = 1.09) increase in IgG4 concentration. In contrast, IgG3 concentration was associated with a 2.77-fold (ß = 1.02) increase with additional reported infections and a 1.75-fold (ß = 0.56) increase with additional vaccinations. For the concentration of IgG1 and IgG2 the increase was similar for vaccinations and infections: 3.16-fold (ß = 1.15) and 3.86-fold (ß = 1.35) for IgG1 and 1.49-fold (ß = 0.4) and 1.8-fold (ß = 0.59) for IgG2. There was no significant association between the PFOA serum concentration group and the concentration of any IgG subclass. Age was not significantly associated with any IgG subclass, while sex had a significant association with IgG2. The number of vaccinations, but not the number of self-reported SARS-CoV-2 infections, was significantly associated with the ratio of IgG4/IgG1 (Table 5). Interaction terms, chronic diseases, medication intake and being a smoker were tested but were not significant. The results shown in Table 5 are supported by the sensitivity analysis using logarithmically transformed PFOA data (Table S3). Furthermore, PFOA was also not significantly associated with any IgG subclass when including days since last SARS-CoV-2 exposure (Table S4).

Similar results were found when including the number of antigen exposures in the model, instead of the number of vaccinations and the number of infections separately. The number of antigen exposures was significantly associated with the logarithmically transformed IgG concentration for each class. However, the number of antigen exposures was no longer significantly associated with the logarithmically transformed IgG4/IgG1 ratio. The PFOA category was not associated with any IgG subclass.

## 4. Discussion

In this study, PFOA serum concentration did not show a significant association with the concentration of any anti-spike IgG subclasses. With regard to PFOA and the antibody formation endpoint in particular, the HBM Commission did not consider the available data to be sufficient at the time and saw a need for further research [52]. In light of this, the 2022 follow-up study examined whether high internal PFOA exposure could be associated with a reduced immune response to vaccination or SARS-CoV-2 infection. Age is a confounder of both the PFOA exposure [47] and the immune system [21]. In addition, as observed in this analysis, age may influence vaccination acceptance and exposure to infection. The number of antigen exposures was the main factor associated with a change in IgG concentration and subclass distribution.

Beyerlein et al. [53] performed a retrospective analysis on the reported COVID-19 cases in the district of Altötting, Bavaria, between 2021 and 2022 and compared the rate of breakthrough infections with the rest of Bavaria. This report shows similar rates of COVID-19 immunisation, seven-day incidence and similar or lower rates of breakthrough infections. After age stratification, there was no difference that would indicate that past PFOA exposure was associated with increased COVID-19 breakthrough rate. The high percentage of participants with positive or highly positive IgG1 and IgG3 titres was consistent with the reported antigen exposures: 92.55% of the participants in our study received at least one SARS-CoV-2 vaccination, and 45.87% had at least one PCR-confirmed SARS-CoV-2 infection at the time of the follow-up study. IgG1 and IgG3 regulate important aspects of the immune system (phagocytosis, apoptosis, etc.). While very high IgG3 titres are maintained only for a short time, IgG1 levels can remain in serum at high concentration for a longer period and is the dominant IgG subclass after viral antigen exposure, binding to the viral surface protein as part of the immune response [7,54,55].

Repeated antigen exposure was associated with an increase in the concentration of IgG4. IgG4 has a lower potential to activate Fc-mediated effector functions compared to IgG1 and IgG3 [56]. Interestingly, the ratio of IgG4/IgG1 was significantly associated with the number of vaccinations, but not with the number of self-reported infections. The higher estimate for the number of vaccinations compared to the number of infections for IgG4, but not for IgG1, supports this conclusion. Nevertheless, there was no evidence that vaccination protection and SARS-CoV-2 neutralisation abilities were adversely affected. Dedicated studies with additional examinations, such as antibody neutralisation activity and different designs, would be necessary.

Similar to the study by Irrgang et al. [21], the ratio of IgG4/IgG1 started to increase after the second vaccination and became IgG4-dominant in 10.25% of the participants with three vaccinations. The same trend, however, does not appear when looking at SARS-CoV-2 infections alone. Other studies also found that vaccination, in particular vaccines based on the mRNA platform are associated with a shift towards IgG4-dominated IgG, and not just repeated antigen exposure [21,25,32]. Most participants in our study had high or very high IgG4 antibody levels after the third vaccination, similar to other studies [21,25]. Although the percentage of participants with negative IgG4 decreased with the increased number of vaccinations, there were still 32.05% (25) among those with two vaccinations and 6.37% (33) among those with three vaccinations negative for IgG4. A shift towards a balanced IgG4/IgG1 ratio or a IgG4 dominated one was already present in the group with two antigen exposures. In total, the ratio was balanced in 10.45%, and IgG4 dominated in 8.27% (53) of the study participants. This is unexpected, as a standard immune response would be for the IgG1 titre to be greatly above that of IgG4 after vaccination [21]. Studies are currently investigating the mechanism behind the high IgG4 titre and its role in the immune response [21,57,58,59]. This highlights the need for further studies and investigations regarding the long-term effects of repeated vaccination or infection with SARS-CoV-2 on the immune system response.

The association between PFOA concentrations < 10 µg/L or ≥10 µg/L in serum, the value determined by the HBM Commission for adverse health effects in the general population (HBM-II), and the immune response was evaluated by focusing on IgG subclasses and on the IgG4 to IgG1 ratio. The PFOA serum concentration group was not associated with a significant change in the distribution of IgG categories for any IgG subclass. Regression analysis showed no significant association between PFOA and any IgG subclass in our study population. The similar rate of breakthrough infections in the PFOA-exposed study region and the rest of Bavaria also points to a similar immune response existing irrespective of PFOA exposure [53]. While Porter et al. [43] have shown some small inverse association between PFOA and SARS-CoV-2 antibodies, this was in the context of occupational exposure to PFAS. Hollister et al. [60] determined that other PFAS (PFOS, PFHxS, PFNA) were significantly associated with a lower peak of anti-S SARS-CoV-2 antibody titres after infection, but PFOA was not associated with altered immune response after infection or vaccination.

One limitation of our data is that this was a cross-sectional study; as such, we could not follow the development of the IgG subclass distribution and overall IgG titre pre- and post-vaccination or infection. A possible source of participation bias may have been introduced as only willing and able participants to a previous PFAS Human Biomonitoring study in the region were included. Further research with a more representative study population and a longitudinal approach could improve the generalizability of the findings and provide arguments for a possible causal relationship or lack thereof. Another limitation is the lack of a timeline and sequence for participants’ infections and vaccinations, allowing for a better modelling of more factors that could affect the anti-S SARS-CoV-2 antibodies titres. Other health factors, such as diseases or medications influencing the anti-S SARS-CoV-2 antibody titres, should be well documented by the study participants. While IgG is a reliable marker for immunity, there are additional immunological aspects that could be considered, such as neutralising antibody activity, characterising T-cells, B-cells and N-297 glycosylation [7,8]. Given the SARS-CoV-2 vaccination campaigns that took place at the time of the study and the high incidence of COVID-19 in the two years before, a relatively small number of participants (18; 2.73%) had no PCR-confirmed infection and no SARS-CoV-2 vaccination. The timing of the study was also advantageous, as we were able to include many participants who had been exposed to the SARS-CoV-2 S-antigen several times. This study is among the few studies examining the effects of PFOA on SARS-CoV-2 antibodies in an adult study population. Furthermore, we were able to provide additional epidemiological data regarding the number of SARS-CoV-2 antigen exposures and IgG subclasses.

## 5. Conclusions

The number of self-reported infections and vaccinations was significantly associated with IgG1 and IgG4 titre categories for the study population. The PFOA serum concentration group was not associated with the concentration of specific IgG subclasses. IgG was dominated by IgG4 for 8.27% (53/641) of the participants, but only among those who had reported three vaccinations. Further research is required to understand the reason for the high IgG4 titre and its implications in the long-term immunity response against SARS-CoV-2.

## Figures and Tables

**Table 1:** Baseline characteristics.

	*n*	Percent
Gender		
Female	377	57.21%
Male	282	42.79%
Age		
18–39	113	17.15%
40–59	304	46.13%
≥60	242	36.72%
PFOA		
<10 µg/L	362	54.93%
≥10 µg/L	297	45.07%
Number of self-reported vaccinations		
0	49	7.45%
1	13	1.98%
2	78	11.85%
3	518	78.72%
Missing	1	
Number of self-reported, PCR-confirmed infections		
0	354	54.13%
1	283	43.27%
2	13	1.99%
3	4	0.61%
Missing	5	
Number of antigen exposures (vaccinations + infections)		
0	18	2.73%
1	30	4.55%
2	37	5.61%
3	368	55.84%
4	197	29.89%
5+	9	1.37%

**Table 2:** Anti-S SARS-CoV-2 IgG subclasses antibody by number of vaccinations.

		Number of Vaccinations				
		0	1	2	3	Sum
IgG1 *n* = 658	Negative	16 (32.65%)	0 (0%)	0 (0%)	2 (0.39%)	18 (2.74%)
	Positive	32 (65.31%)	11 (84.62%)	41 (52.56%)	269 (51.93%)	353 (53.65%)
	Highly positive	1 (2.04%)	2 (15.38%)	37 (47.44%)	247 (47.68%)	287 (43.62%)
IgG2 *n* = 658	Negative	1 (2.04%)	0 (0%)	0 (0%)	0 (0%)	1 (0.15%)
	Positive	48 (97.96%)	13 (100%)	75 (96.15%)	484 (93.44%)	620 (94.22%)
	Highly positive	0 (0%)	0 (0%)	3 (3.85%)	34 (6.56%)	37 (5.62%)
IgG3 *n* = 658	Negative	37 (75.51%)	8 (61.54%)	23 (29.49%)	159 (30.69%)	227 (34,5%)
	Positive	12 (24.49%)	5 (38.46%)	55 (70.51%)	359 (69.31%)	431 (65,5%)
	Highly positive	0 (0%)	0 (0%)	0 (0%)	0 (0%)	0 (0%)
IgG4 *n* = 658	Negative	47 (95.92%)	8 (61.54%)	25 (32.05%)	33 (6.37%)	113 (17.17%)
	Positive	2 (4.08%)	4 (30.77%)	50 (64.1%)	410 (79.15%)	466 (70.82%)
	Highly positive	0 (0%)	1 (7.69%)	3 (3.85%)	75 (14.48%)	79 (12.01%)
Ratio IgG4/IgG1 *n* = 641	IgG1-dominated	33 (100%)	12 (92.31%)	75 (96.15%)	401 (77.56%)	521 (81.28%)
	Balanced	0 (0%)	1 (7.69%)	3 (3.85%)	63 (12.19%)	67 (10.45%)
	IgG4-dominated	0 (0%)	0 (0%)	0 (0%)	53 (10.25%)	53 (8.27%)

**Table 3:** Anti-S SARS-CoV-2 IgG antibody by number of self-reported PCR-confirmed SARS-CoV-2 infections.

		Number of Self-Reported PCR-Confirmed SARS-CoV-2 Infections				
		0	1	2	3	Sum
IgG1 *n* = 654	Negative	13 (3.67%)	4 (1.41%)	0 (0%)	0 (0%)	17 (2.6%)
	Positive	241 (68.08%)	104 (36.75%)	5 (38.46%)	1 (25%)	351 (53.67%)
	Highly positive	100 (28.25%)	175 (61.84%)	8 (61.54%)	3 (75%)	286 (43.73%)
IgG2 *n* = 654	Negative	1 (0.28%)	0 (0%)	0 (0%)	0 (0%)	1 (0.15%)
	Positive	342 (96.61%)	257 (90.81%)	13 (100%)	4 (100%)	616 (94.19%)
	Highly positive	11 (3.11%)	26 (9.19%)	0 (0%)	0 (0%)	37 (5.66%)
IgG3 *n* = 654	Negative	164 (46.33%)	55 (19.43%)	5 (38.46%)	1 (25%)	225 (34.4%)
	Positive	190 (53.67%)	228 (80.57%)	8 (61.54%)	3 (75%)	429 (65.6%)
	Highly positive	0 (0%)	0 (0%)	0 (0%)	0 (0%)	0 (0%)
IgG4 *n* = 654	Negative	58 (16.38%)	47 (16.61%)	7 (53.85%)	0 (0%)	112 (17.13%)
	Positive	273 (77.12%)	181 (63.96%)	5 (38.46%)	4 (100%)	463 (70.8%)
	Highly positive	23 (6.5%)	55 (19.43%)	1 (7.69%)	0 (0%)	79 (12.08%)
Ratio IgG4/IgG1 *n* = 638	IgG1-dominated	271 (79.24%)	231 (82.8%)	12 (92.31%)	4 (100%)	518 (81.19%)
	Balanced	43 (12.57%)	23 (8.24%)	1 (7.69%)	0 (0%)	67 (10.5%)
	IgG4-dominated	28 (8.19%)	25 (8.96%)	0 (0%)	0 (0%)	53(8.31%)

**Table 4:** Anti-S SARS-CoV-2 IgG subclasses by number of SARS-CoV-2 antigen exposures (vaccinations and infections).

		Number of SARS-CoV-2 Antigen Exposures					
		0	1	2	3	4	5+
IgG1 *n* = 659	Negative	14 (77.78%)	3 (10%)	0 (0%)	1 (0.27%)	1 (0.51%)	0 (0%)
	Positive	4 (22.22%)	27 (90%)	30 (81.08%)	240 (65.22%)	50 (25.38%)	2 (22.22%)
	Highly positive	0 (0%)	0 (0%)	7 (18.92%)	127 (34.51%)	146 (74.11%)	7 (77.78%)
IgG2 *n* = 659	Negative	1 (5.56%)	0 (0%)	0 (0%)	0 (0%)	0 (0%)	0 (0%)
	Positive	17 (94.44%)	30 (100%)	37 (100%)	354 (96.2%)	174 (88.32%)	9 (100%)
	Highly positive	0 (0%)	0 (0%)	0 (0%)	14 (3.8%)	23 (11.68%)	0 (0%)
IgG3 *n* = 659	Negative	17 (94.44%)	19 (63.33%)	21 (56.76%)	148 (40.22%)	21 (10.66%)	2 (22.22%)
	Positive	1 (5.56%)	11 (36.67%)	16 (43.24%)	220 (59.78%)	176 (89.34%)	7 (77.78%)
	Highly positive	0 (0%)	0 (0%)	0 (0%)	0 (0%)	0 (0%)	0 (0%)
IgG4 *n* = 659	Negative	18 (100%)	26 (86.67%)	26 (70.27%)	37 (10.05%)	6 (3.05%)	1 (11.11%)
	Positive	0 (0%)	4 (13.33%)	10 (27.03%)	305 (82.88%)	140 (71.07%)	7 (77.78%)
	Highly positive	0 (0%)	0 (0%)	1 (2.70%)	26 (7.07%)	51 (25.89%)	1 (11.11%)
Ratio Ig4/IgG1 *n* = 641	IgG1-dominated	4 (100%)	27 (100%)	35 (94.59%)	296 (80.43%)	151 (77.04%)	8 (88.89%)
	Balanced	0 (0%)	0 (0%)	2 (5.41%)	44 (11.96%)	20 (10.2%)	1 (11.11%)
	IgG4-dominated	0 (0%)	0(0%)	0 (0%)	28 (7.61%)	25 (12.76%)	0 (0%)

**Table 5:** Generalised linear regression results for anti-S SARS-CoV-2 IgG subclasses (*n* = 653).

IgG	Effect		Estimate (ß)	95% CI	*p* Value
IgG1					
	Number of vaccinations		1.15	1.01–1.29	<0.0001
	Number of infections		1.35	1.14–1.56	<0.0001
	PFOA (≥10 µg/L)	Ref. < 10 µg/L	0.09	(−0.15)–0.33	0.461
	Age (40–59)	Ref. < 40	0.11	(−0.22)–0.44	0.508
	Age (≥60)	Ref. < 40	0.06	(−0.29)–0.42	0.723
	Sex (male)	Ref. female	0.08	(−0.15)–0.31	0.510
IgG2					
	Number of vaccinations		0.4	0.28–0.52	<0.0001
	Number of infections		0.59	0.41–0.77	<0.0001
	PFOA (≥10 µg/L)	Ref. < 10 µg/L	−0.19	(−0.4)–0.01	0.06
	Age (40–59)	Ref. < 40	−0.18	(−0.46)–0.1	0.203
	Age (≥60)	Ref. < 40	−0.05	(−0.35)–0.25	0.746
	Sex (male)	Ref. female	0.2	0.003–0.4	0.046
IgG3					
	Number of vaccinations		0.56	0.42–0.69	<0.0001
	Number of infections		1.02	0.82–1.22	<0.0001
	PFOA (≥10 µg/L)	Ref. < 10 µg/L	−0.1	(−0.32)–0.13	0.408
	Age (40–59)	Ref. < 40	−0.23	(−0.54)–0.08	0.141
	Age (≥60)	Ref. < 40	−0.11	(−0.45)–0.23	0.516
	Sex (male)	Ref. female	0.11	(−0.11)–0.32	0.346
IgG4					
	Number of vaccinations		1.76	1.54–1.97	<0.0001
	Number of infections		1.09	0.76–1.42	<0.0001
	PFOA (≥10 µg/L)	Ref. < 10 µg/L	−0.1	(−0.48)–0.28	0.594
	Age (40–59)	Ref. < 40	−0.12	(−0.64)–0.39	0.646
	Age (≥60)	Ref. < 40	−0.17	(−0.74)–0.39	0.543
	Sex (male)	Ref. female	0.37	0.004–0.74	0.048
IgG4/IgG1 (*n* = 541)					
	Number of vaccinations		0.79	0.26–1.31	0.003
	Number of infections		−0.20	(−0.57)–0.16	0.278
	PFOA (≥10 µg/L)	Ref. < 10 µg/L	−0.13	(−0.55)–0.28	0.522
	Age (40–59)	Ref. < 40	−0.39	(−0.95)–0.17	0.174
	Age (≥60)	Ref. < 40	−0.14	(−0.75)–0.47	0.645
	Sex (male)	Ref. female	0.26	(−0.13)–0.66	0.193

## Data Availability

The data evaluated for this study is not publicly available as it includes confidential personal medical data, and their distribution and use has been restricted in the study participants’ agreement.

## Supplementary Materials

The following supporting information can be downloaded at https://www.mdpi.com/article/10.3390/toxics14030192/s1. Table S1: Number of SARS-CoV-2 vaccines by age group; Table S2: Number of self-reported, PCR confirmed SARS-CoV-2 infections by age group; Table S3: Generalised linear regression results for anti-S SARS-CoV-2 IgG subclasses with log-transformed PFOA as independent variable; Table S4: Generalised linear regression results for anti-S SARS-CoV-2 IgG subclasses with log-transformed PFOA and days since last SARS-CoV-2 exposure as independent variables.

## Abbreviations

The following abbreviations are used in this manuscript:

ADCP	Antibody-dependent cellular phagocytosis
HBM	Human BioMonitoring
IgG	Immunoglobulin G
PFAS	Perfluoroalkyl substance
PFHxS	Perfluorohexane sulfonic acid
PFNA	Perfluorononanoic acid
PFOA	Perfluorooctanoic acid
PFOS	Perfluorooctane sulfonic acid
Ref.	Reference category
