# Supplementary material for: SARS-CoV-2 Anti-Spike IgG Subclass Titres in a Population with Prior Exposure to Perfluorooctanoic Acid (PFOA)
Source: Toxics. 2026 Feb 25;14(3):192. doi: 10.3390/toxics14030192 (PMC13030776; doi:10.3390/toxics14030192)
Supplement: Supplementary file 1 [file toxics-14-00192-s001.zip › toxics-4103553-supplementary.pdf]

Supplemental tables

Table S1. Number of SARS-CoV-2 vaccines by age group

Age	Number of self-reported vaccinations				
	**0**	**1**	**2**	**3**	**Total**
18–39	7 (6.19%)	1 (0.88%)	30 (26.55%)	75 (66.37%)	113
40–59	28 (9.24%)	6 (1.98%)	34 (11.22%)	235 (77.56%)	303
≥60	14 (5.79%)	6 (2.48%)	14 (5.79%)	208 (85.95%)	242
Total	49	13	78	518	658

Table S2. Number of self-reported, PCR confirmed SARS-CoV-2 infections by age group

Age	Number of self-reported infections				
	**0**	**1**	**2**	**3**	**Total**
18–39	34 (30.09%)	74 (65.49%)	4 (3.54%)	1 (0.88%)	113
40–59	152 (50.217)	144 (47.52%)	5 (1.65%)	2 (0.66%)	303
≥60	168 (70.59%)	65 (27.31%)	4 (1.68%)	1 (0.42%)	238
Total	354	283	13	4	654

Table S3. Generalized linear regression results for anti-S SARS-CoV-2 IgG subclasses with log-transformed PFOA as independent variable. (n = 653)

IgG	Effect		Estimate(ß)	95% CI	p value
IgG1					
	Number of vaccinations		1.15	1.01–1.29	<0.0001
	Number of infections		1.35	1.14–1.56	<0.0001
	Log (PFOA)		0.04	(−0.11)–0.19	0.617
	Age (40–59)	Ref. < 40	0.11	(−0.22)–0.44	0.522
	Age (≥60)	Ref. < 40	0.07	(−0.31)–0.44	0.730
	Sex (male)	Ref. female	0.08	(−0.16)–0.31	0.524
IgG2					
	Number of vaccinations		0.4	0.29–0.52	<0.0001
	Number of infections		0.59	0.41–0.77	<0.0001
	Log (PFOA)		−0.04	(−0.17)–0.09	0.534
	Age (40–59)	Ref. < 40	−0.19	(−0.48)–0.09	0.174
	Age (≥60)	Ref. < 40	−0.09	(−0.40)–0.22	0.573
	Sex (male)	Ref. female	0.19	0.007–0.39	0.059
IgG3					
	Number of vaccinations		0.56	0.43–0.69	<0.0001
	Number of infections		1.02	0.82–1.21	<0.0001
	Log (PFOA)		−0.02	(−0.16)–0.13	0.815
	Age (40–59)	Ref. < 40	−0.24	(−0.55)–0.07	0.132
	Age (≥60)	Ref. < 40	−0.13	(−0.48)–0.22	0.452
	Sex (male)	Ref. female	0.10	(−0.12)–0.32	0.373
IgG4					
	Number of vaccinations		1.76	1.54–1.98	<0.0001
	Number of infections		1.08	0.75–1.42	<0.0001
	Log (PFOA)		0.07	(−0.17)–0.30	0.585

	Age (40–59)	Ref. < 40	−0.17	(−0.69)–0.35	0.526
	Age (≥60)	Ref. < 40	−0.28	(−0.86)–0.31	0.355
	Sex (male)	Ref. female	0.34	(−0.03)–0.71	0.07
IgG4/IgG1 (*n* = 541)					
	Number of vaccinations		0.79	0.26–1.31	0.003
	Number of infections		−0.20	(−0.57)–0.17	0.281
	Log (PFOA)		−0.03	(−0.29)–0.23	0.827
	Age (40–59)	Ref. < 40	−0.40	(−0.96)–0.17	0.171
	Age (≥60)	Ref. < 40	−0.17	(−0.80)–0.46	0.603
	Sex (male)	Ref. female	0.26	(−0.14)–0.66	0.205

Table S4. Generalized linear regression results for anti-S SARS-CoV-2 IgG subclasses with log-transformed PFOA and days since last SARS-CoV-2 exposure as independent variables. (*n* = 611)

IgG	Effect		Estimate(ß)	95% CI	*p* value
IgG1					
	Number of vaccinations		1.09	0.90–1.27	<0.0001
	Number of infections		1.20	0.95–1.46	<0.0001
	Log (PFOA)		0.05	(−0.11)–0.21	0.544
	Age (40–59)	Ref. < 40	0.05	(−0.29)–0.39	0.764
	Age (≥60)	Ref. < 40	0.04	(−0.34)–0.43	0.821
	Sex (male)	Ref. female	0.04	(−0.21)–0.28	0.766
	Days since last SARS-CoV-2 exposure		−0.002	(−0.004)–(−0.00001)	0.048
IgG2					
	Number of vaccinations		0.39	0.23–0.54	<0.0001
	Number of infections		0.47	0.25–0.69	<0.0001
	Log (PFOA)		−0.02	(−0.15)–0.12	0.815
	Age (40–59)	Ref. < 40	−0.22	(−0.51)–0.07	0.133
	Age (≥60)	Ref. < 40	−0.09	(−0.42)–0.24	0.576
	Sex (male)	Ref. female	0.14	(−0.06)–0.35	0.178
	Days since last SARS-CoV-2 exposure		−0.002	(−0.004)–(−0.0007)	0.005
IgG3					
	Number of vaccinations		0.66	0.49–0.83	<0.0001
	Number of infections		1.003	0.76–1.24	<0.0001
	Log (PFOA)		0.03	(−0.12)–0.18	0.692
	Age (40–59)	Ref. < 40	−0.27	(−0.59)–0.05	0.101
	Age (≥60)	Ref. < 40	−0.19	(−0.55)–0.17	0.305
	Sex (male)	Ref. female	0.009	(−0.22)–0.24	0.942
	Days since last SARS-CoV-2 exposure		−0.002	(−0.004)–(−0.000005)	0.049
IgG4					
	Number of vaccinations		1.90	1.61–2.19	<0.0001
	Number of infections		1.002	0.60–1.41	<0.0001
	Log (PFOA)		0.14	(−0.11)–0.39	0.271
	Age (40–59)	Ref. < 40	−0.21	(−0.74)–0.33	0.448
	Age (≥60)	Ref. < 40	−0.33	(−0.94)–0.27	0.281
	Sex (male)	Ref. female	0.24	(−0.15)–0.62	0.222
	Days since last SARS-CoV-2 exposure		−0.004	(−0.006)–(−0.0002)	0.035

IgG4/IgG1 (*n* = 522)				
Number of vaccinations		0.88	0.21–1.54	0.0095
Number of infections		−0.25	(−0.70)–0.20	0.270
Log (PFOA)		−0.03	(−0.30)–0.23	0.816
Age (40–59)	Ref. < 40	−0.42	(−1.00)–0.16	0.154
Age (≥60)	Ref. < 40	−0.16	(−0.82)–0.50	0.627
Sex (male)	Ref. female	0.25	(−0.16)–0.66	0.232
Days since last SARS-CoV-2 exposure		−0.001	(−0.005)–(0.003)	0.587
